# Evaluating hypothetical interventions effects on hospital-acquired infection outcomes with stacked probability visualization: R Shiny apps based on a multistate modelling approach

**DOI:** 10.1371/journal.pone.0343837

**Published:** 2026-03-16

**Authors:** Jean-Pierre Gnimatin, Marlon Grodd, Susanne Weber, Derek Hazard, Martin Wolkewitz

**Affiliations:** 1 Institute of Medical Biometry and Statistics, Faculty of Medicine and Medical Center, University of Freiburg, Freiburg, Germany; 2 Freiburg Center for Data Analysis, Modeling and AI, University of Freiburg, Freiburg, Germany; Sapienza University of Rome: Universita degli Studi di Roma La Sapienza, ITALY

## Abstract

**Background:**

Hospital-acquired infections (HAIs), contribute to increased morbidity, prolonged hospital stays, and higher healthcare costs. Evaluating intervention effects in dynamic clinical settings requires advanced modeling techniques. This study presents HAISim and StaViC, two interactive R Shiny apps designed to support decision-making in Infection Prevention and Control.

**Methods:**

A six-state extended illness–death multistate model was built with a time-constant transition hazard assumption. The multistate model maps patient trajectories involving healthcare-associated infections as an intermediate event, and mortality and discharge as absorbing events in order to describe the time-dependent dynamics within hospitals. Using two settings, we simulated the implementation of hypothetical treatments by modifying hazard rates: Setting 1 (improved treatment intervention only) and Setting 2 (combined enhanced treatment and infection prevention). These were used to create the interactive and user-friendly R Shiny Apps HAISim (HAIs Interventions Simulator) and StaViC (Stacked probAbility Visualization & Comparison). The Shiny Apps use inputs from literature or user data, such as transition-specific hazard rates and intervention-related parameters.

**Results:**

HAISim models the effects of hypothetically improved treatment and infection prevention on outcomes such as the number of lives saved and the number of patient days decreased by simulating a hypothetical scenario based on actual clinical data. StaViC makes it possible to compare potential interventions and their impacts before and after implementation by visualizing the stacked probabilities of patients across various health conditions.

**Conclusions:**

These tools bridge methodological rigor and practical implementation, offering hospitals a flexible framework to prioritize cost-effective IPC strategies.

## Introduction

Infection Prevention and Control (IPC) is of major importance in healthcare and community settings across the globe [1,2]. Due to the special interest in their implication in care settings where there is a population mainly made of people suffering from different conditions, ranging from accidents, diseases, or infections and where patients may face different competing events such as death, aggravation of conditions, discharge, etc. [3–5]. Analyzing the effect of IPC programs already in place or new ones to be implemented is therefore of great importance. Multistate models have been extensively used in such settings where patients can transition between different disease states over time, to investigate several infections such as ventilator-associated pneumonia (VAP), bloodstream infections (BSIs), urinary tract infections (UTI), lower respiratory tract infections (LRTIs), etc. [6–9]. While multistate modeling offers a robust framework in analyzing complex disease progression, capturing patient transitions and provide valuable insights of potential interventions on hazards; its traditional statistical outputs often lack accessibility for decision-makers, including clinicians, hospital administrators, and policymakers. The integration of interactive visualization tools, such as R Shiny applications, can bridge this gap by transforming complex model outputs into intuitive, policy-relevant insights. In this study, we introduce two interactive and user-friendly R Shiny Apps: HAISim (Hospital-Acquired infection Interventions Simulator) and StaViC (Stacked probAbility Visualization & Comparison) designed to support decision-making in IPC. Which of a given set of treatments or interventions will not only have the best impact on clinical outcomes but will also have optimal effects on hospital length of stay or overall healthcare expenses? In how many days could a patient reach the peak of the infection state or the peak probability of death? Those are example of questions, relevant to clinicians but also to healthcare deciders, where the Shiny Apps from this study could be of support for simulations purposes. Our goal is to provide stakeholders at different levels of healthcare decision-making with robust analytical tools to evaluate and compare interventions.

## Materials and methods

### Multistate model formulation

**Fig 1:**
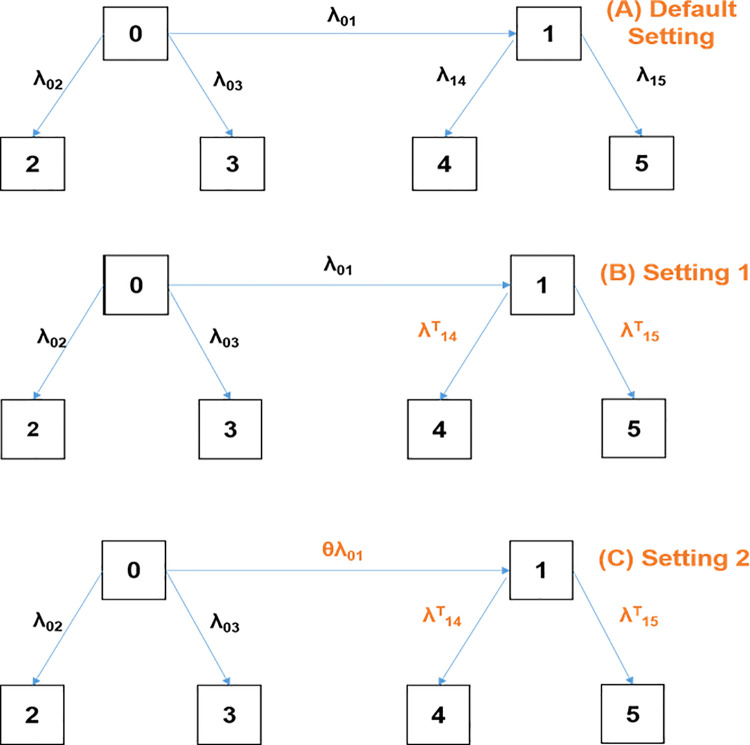
Multi state model formulation and defined settings. ***(A)Default setting*** – Extended illness-death model, ***(B)* *Setting 1*-** Default setting + enhanced treatment (only Intervention 1), ***(C)*** ***Setting 2***- Default setting + enhanced treatment and infection prevention (combination of interventions 1 and 2). Arrows denote direction of possible transitions; 0-Admission into hospital, *1*- Hospital-acquired infections (HAI), *2*-Discharged alive after Admission (and stay in hospital setting without acquiring HAI), *3*-Death after Admission (and stay in hospital setting without acquiring HAI), *4*-Discharged alive after admission followed by HAI, *5-*Death after Admission followed by HAI, *λ*_*ij*_ – Transition hazard rate from state *i* to another sate *j*, *λ*^*T*^_*ij*_ – Transition hazard rate from state *i* to another sate *j* in the context of enhanced treatment, *θ* – Disease prevention factor.

The study builds on the extended illness-death model, considering infection as an intermediate state during hospitalization, with hospital death or discharge alive as absorbing states. The model includes 6 states (See **Fig 1**) as follows: 0-Admission into hospital, 1-Hospital-acquired infections (HAI), 2-Discharged Alive after Admission (and stay in hospital setting without acquiring HAI), 3-Death after Admission (and stay in hospital setting without acquiring HAI), 4-Discharged Alive after admission followed by HAI, 5-hospital Death after Admission followed by HAI. Moving from a state *i* to another state *j* is done with the specific-transition hazard rate *λ*_*ij*_. The model assumes the Markov property in which the future clinical course depends only on the current infection state and not on past states [10], an assumption which has been used in hospital-acquired infection modelling [11]. The model assumes a homogeneous population, resulting in marginal transition hazards that summarize overall patient risk despite potential unobserved heterogeneity [12]. The last assumption is that patients enter the hospital with no prior infection and the infection is only acquired after admission into the healthcare setting. We denote as **Default setting**, the model with the characteristics as previously described. We assume two (2) interventions: first, we assumed the introduction of a new treatment option that leads to enhanced treatment of patients (Intervention 1), reducing mortality rates and increasing the number of patients who recover and are discharged alive after HAI. Second, we assume an infection prevention strategy (Intervention 2) which was previously introduced by Hazard *et al.* [13]. We denote as **Setting 1**, the model where the default setting is extended with intervention 1 (ONLY ENHANCED TREATMENT). We denote as **Setting 2**, the model where both intervention 1 and intervention 2 are being simultaneously implemented (ENHANCED TREATMENT + IMPROVED INFECTION PREVENTION).

Implementing Intervention 1, which focuses on enhanced treatment of hospital-acquired infections (HAIs), introduces new transition hazard rates *λ*^*T*^_*ij*_, being transition hazard rate from state *i* to another sate *j* in the context of enhanced treatment (applicable in both Setting 1 & Setting 2). In contrast, implementing intervention 2 introduces *θ* which represents the infection prevention factor which applies exclusively in Setting 2.

We therefore have as described below in Table 1, the following total transmission specific hazard rates:

**Table 1:** Total transition-specific hazard rates for each setting.

	Total transition-specific hazard rates in state 0 (Non-infected)	Total transition-specific hazard rates in state 1 (Infected)
**Default setting**	λ_0_ = λ_01_ + λ_02_ + λ_03_	λ_1_ = λ_14_ + λ_15_.
**Setting 1**	λ_0_ = λ_01_ + λ_02_ + λ_03_	λ_1_ = λ^T^_14_ + λ^T^_15_
**Setting 2**	λ_0_ = θλ_01_ + λ_02_ + λ_03_	λ_1_ = λ^T^_14_ + λ^T^_15_

Where we assumed *λ*^*T*^_*14*_ = *α*(*λ*_*14*_ – *λ*_*02*_) + *λ*_*02*_ and *λ*^*T*^_*15*_ = *β*(*λ*_*15*_ - *λ*_*03*_) + *λ*_*03*_; with enhanced treatment related factors, *α* and *β* Є [0, 1], *α* is the effect of the enhanced treatment on discharge and *β*, on death. We also assumed *θ*, the infection prevention factor varying between [0, 1]. The lower the value of *θ*, the more effective the prevention is at limiting HAIs and similarly, the lower the value of *α* and *β*, the higher the treatment effect.

This means that a value of 0 translates into a maximum effect and a value of 1 translates into no effect for both the enhanced treatment and prevention related factors. (For example, if *α* = 0 in the expression of *λ*^*T*^_*14*_ then, we have *λ*^*T*^_*14*_ hazard of being discharge alive among infected patients being equal to λ_02_, the hazard of being discharge alive among non-infected patients).

### Estimations

We considered both the probability of mortality due to the initial condition that led to hospitalization and the additional mortality risk following the acquisition of an HAI (increasing the hazard of mortality). Furthermore, the HAI was expected to prolong the hospital stay for affected patients, reducing the hazard of discharge. To estimate the reduction in burden, we compared outcomes between the default setting and the alternative intervention settings (1 or 2).

### Estimation of reduced burden from Setting 1 (only Intervention 1)

#### Reduced HAI Mortality: RM_1_.

To determine the reduced mortality (RM_1_) in Setting 1, we calculated the difference between the overall probability of hospital death in the Default Setting (no intervention) denoted Prob 0, and the overall probability in Setting 1(with only intervention 1) denoted Prob 1.

$Prob\text{0}=\frac{\lambda_{\text{1}}\lambda_{\text{0}\text{3}}+\;\lambda_{\text{0}\text{1}}\lambda_{\text{1}\text{5}}}{\lambda_{\text{0}}\lambda_{\text{1}}}$ and $Prob\text{1}=\frac{\lambda_{\text{1}}^{T}\lambda_{\text{0}\text{3}}+\;\lambda_{\text{0}\text{1}}\lambda_{\text{1}\text{5}}^{T}}{\lambda_{\text{0}}\lambda_{\text{1}}^{T}}$ Then, the reduced mortality (RM_1_) is given by:


$${\text{RM}}_{\text{1}}=\text{ Prob }\text{0}\;-\text{ Prob }\text{1}\;=\frac{\lambda_{\text{1}}\lambda_{\text{0}\text{3}}+\;\lambda_{\text{0}\text{1}}\lambda_{\text{1}\text{5}}}{\lambda_{\text{0}}\lambda_{\text{1}}}-\frac{\lambda_{\text{1}}^{T}\lambda_{\text{0}\text{3}}+\;\lambda_{\text{0}\text{1}}\lambda_{\text{1}\text{5}}^{T}}{\lambda_{\text{0}}\lambda_{\text{1}}^{T}}$$ (1)

#### Change in overall Length of stay cLoS_1_.

Similarly, we determined the change in overall length of hospital stay (cLoS_1_) due to intervention 1, by evaluating the difference between the lengths of hospital stay in the Default


$$\text{Setting VS Setting }\text{1};\text{ cLo}{\text{S}}_{\text{1}}\;=\text{ Lo}{\text{S}}_{\text{0}}\;-\text{ Lo}{\text{S}}_{\text{1}}\;=\;\frac{\lambda_{\text{1}}+\;\lambda_{\text{0}\text{1}}}{\lambda_{\text{0}}\lambda_{\text{1}}}-\;\frac{\lambda_{\text{1}}^{T}+\;\lambda_{\text{0}\text{1}}}{\lambda_{\text{0}}\lambda_{\text{1}}^{T}}$$ (2)

#### Estimation of reduction (Number of lives saved and patient-days).

The number of patient-days reduced as well as number of lives saved after implementation of intervention1 is determined by multiplying the previously determined reduced mortality and change in length of hospital stay by the number of patients (*n*). We therefore have the following:


$$\text{Patient}-\text{Days}\text{1}\;={\text{ cLoS}}_{\text{1}}\;*\;n$$ (3)

and


$$\text{LivSaved}\text{1}\;={\text{RM}}_{\text{1}}*n\;$$ (4)

### Estimation of reduced burden from Setting 2 (combination of intervention 1 & intervention 2)

With the purpose of having optimal effects in improving patients’ outcomes, we also simulated a setting where we have the combination of both intervention 1 and 2, translating into both prevention as well as enhanced treatment as shown in Setting 2.

#### Reduced HAI Mortality: RM_2_.

The reduced overall mortality (RM_2_) due to the combination of intervention 1 and intervention 2, is determined by the following: $Prob\text{0}=\frac{\lambda_{\text{1}}\lambda_{\text{0}\text{3}}+\;\lambda_{\text{0}\text{1}}\lambda_{\text{1}\text{5}}}{\lambda_{\text{0}}\lambda_{\text{1}}}$ and $Prob\text{2}=\frac{\lambda_{\text{1}}^{T}\lambda_{\text{0}\text{3}}+\;{\theta \lambda }_{\text{0}\text{1}}\lambda_{\text{1}\text{5}}^{T}}{\lambda_{\text{0}}\lambda_{\text{1}}^{T}}$ Then, the reduced overall mortality (RM_2_) is given by:


$${\text{RM}}_{\text{2}}\;=\text{ Prob }\text{0}\;-\text{ Prob }\text{2}=\;\frac{\lambda_{\text{1}}^{T}\lambda_{\text{0}\text{1}}\lambda_{\text{1}\text{5}}\;-\;{\;\theta \lambda }_{\text{0}\text{1}}\lambda_{\text{1}}\lambda_{\text{1}\text{5}}^{T}}{\lambda_{\text{0}}\lambda_{\text{1}}\lambda_{\text{1}}^{T}}$$ (5)

#### Change in overall Length of stay cLoS_2_.

We proceed to the difference between the lengths of hospital stay in Default setting versus Setting 2. We have:


$$\text{cLo}{\text{S}}_{\text{2}}\;=\text{ Lo}{\text{S}}_{\text{0}}\;-\text{ Lo}{\text{S}}_{\text{2}}\;=\;\frac{\lambda_{\text{1}}+\;\lambda_{\text{0}\text{1}}}{\lambda_{\text{0}}\lambda_{\text{1}}}-\;\frac{\lambda_{\text{1}}^{T}+\;\theta \lambda_{\text{0}\text{1}}}{\lambda_{\text{0}}\lambda_{\text{1}}^{T}}$$ (6)

#### Estimation of reduction (Number of lives saved and reduced patient-days).

Similarly to how it was previously calculated, we therefore have the following:


$$\text{Patient}-\text{Days}\text{2}\;=\;{\text{cLoS}}_{\text{2}}\;*\;n$$ (7)

and


$$\text{LivSaved}\text{2}\;=\;{\text{RM}}_{\text{2}}\;*\;n$$ (8)

A summarized explanation of notations in equations 1–8 is provided in S1 File.

### Transition probabilities

In a multistate model, patients transition between different health states over time. These transitions are governed by a transition probability matrix. The models in this study are Markov models and with the assumption of constant-hazard rates, we can have the mathematical forms of the transition probabilities. The probability to be in state *j* at time *t* while previously being in state *i* at time *s* can be written as: *P*_*ij*_ *(s,t) = P(X(t) = j | X(s)=i)*.

For a model with n states, the transition Probability Matrix is:


$$P=\;(\begin{array}{c} \text{P11}(\text{s},\text{t})\text{ P12}(\text{s},\text{t})\cdots \text{ P1n}(\text{s},\text{t})\\ \text{P21}(\text{s},\text{t})\text{ P22}(\text{s},\text{t})\cdots \text{ P2n}(\text{s},\text{t})\\ \vdots \;\vdots \;\ddots \;\vdots \;\\ \text{Pn1}(\text{s},\text{t})\text{ Pn2}(\text{s},\text{t})\cdots \text{ Pnn}(\text{s},\text{t}) \end{array})$$

Stacked probability plots are graphical representations of the state occupation probabilities over time. They help visualize the probability of being in different states of the model over time. These plots help in understanding disease progression dynamics over a given time period and also for comparing interventions by illustrating how interventions shift probabilities across states. The mathematical forms for the Transition probability matrix and state specific probabilities for Setting 1 and Setting 2 are available in S2 File. A more detailed approach for determining the transition probabilities in an extended illness-death model under the assumption of constant hazard rates can be found in the work of von Cube et al. [11]

### R Shiny apps presentation

Based on the previously described methodology, two (2) interactives and user-friendly R Shiny apps were developed for simulations, accessible at the following links:HAISim (https://haisim.imbi.uni-freiburg.de/) and StaViC (https://stavic.imbi.uni-freiburg.de/). The R code used to implement the Shiny apps is freely available on GitHub and can be accessed through the following repositories: https://github.com/JP-GMT/HAISim and https://github.com/JP-GMT/StaViC.

While the multistate model underlying the Shiny apps does not currently account for recurrent infections, the concept can be extended to include them to address other specific research objectives. The Shiny apps can then be adapted accordingly, since the source code is freely available in the repository for modification.

### Hospital-Acquired infection Interventions Simulator (HAISim) Shiny App

**Fig 2:**
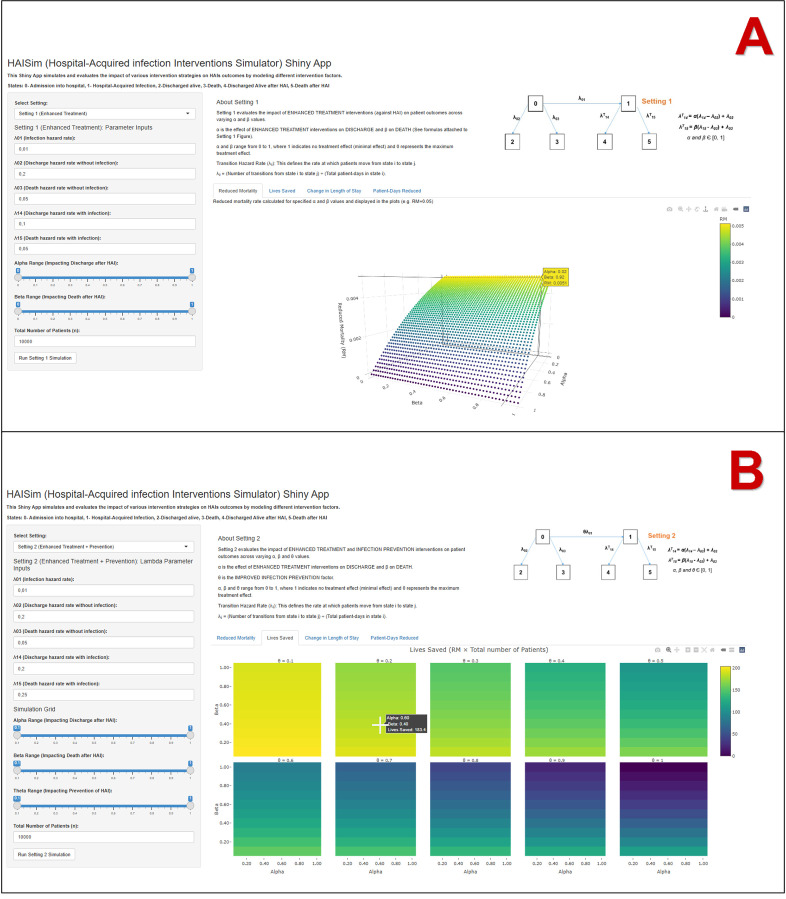
Overview of the interfaces of the HAISim Shiny App. A-Interface of HAISim based on Setting 1(Enhanced treatment as the sole intervention); B-Interface of the HAISim based on Setting 2 (Combination of enhanced treatment and prevention strategies). HAISim simulates and evaluates the impact of various intervention strategies on hospital-acquired infections outcomes by modeling different intervention factors.

The HAISim Shiny App, as seen in Fig 2, can be used for simulations mimicking Setting 1(Enhanced treatment) or Setting 2 (Enhanced treatment + Infection prevention) to assess the impacts on key outcomes such as mortality, lives saved, changes in length of stay, and reductions in patient-days. In the sidebar panel on the App, the user has the option to select the Setting of interest (1 or 2), then comes the input of the values of *λ*, where the user needs to enter the values of each transition hazard. The last thing to take into consideration is the number of patients (by default n = 10000).

The interactive nature of the application allows users to explore the generated plots dynamically. By hovering the cursor over the plot, users can view the corresponding values for specific points. Additionally, the HAISim (based on Setting 1) supports zooming in and out and 360° rotation of the output-plot to examine it from different angles, providing a comprehensive and flexible visualization experience. To proceed with the simulations, it is essential to recall the calculation of a transition hazard rate *λ*_*ij*_ from a state *i* to another *j* is calculated by


$$\lambda ij=\frac{\text{Number of patients who transited from state i to state j}}{\text{Sum of patient}-\text{days in the state i}}$$

A practical demonstration of these calculations using nosocomial infection data has been previously conducted by Wolkewitz et al. [14]

### Stacked probAbility Visualization & Comparison (StaViC) Shiny App

**Fig 3:**
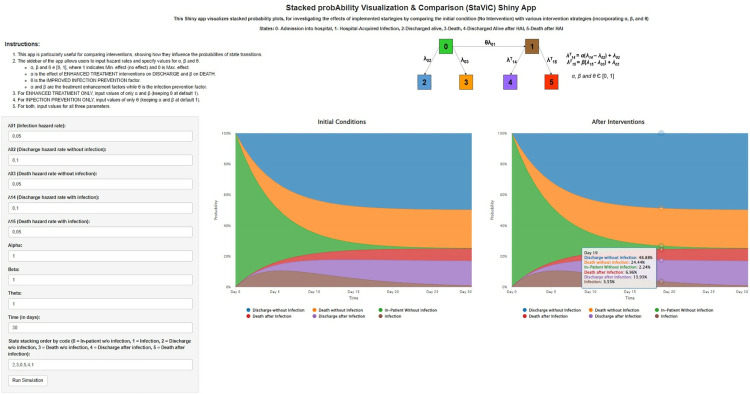
Overview of the interfaces of the StaViC Shiny app. *Initial condition (Left plot) vs. After intervention (Right plot). StaViC helps to understand the dynamics of patients who acquire an infection after hospital admission,* *through the visualization of* *the probability of patients being in different health states of the model over time.*

StaViC was designed to help visualize the probability of patients being in different states of the model over time (Fig 3). It helps to understand the dynamics of patients who acquire an infection after hospital admission. This app is particularly useful for comparing interventions, showing how they influence the probabilities of state transitions, as described under the subsection Transition probabilities (of the Materials and methods section)of this publication. The sidebar of the app allows users to input hazard rates and specify values for *α*, *β* and *θ*. Let’s recall that a value of 1 for each of these parameters indicates no effect, while a value of 0 represents the maximum effect. For simulation under Setting 1 (enhanced treatment only), users should enter values for *α* and *β*, leaving *θ* at its default value of 1 (no effect). For simulation under Setting 2 (enhanced treatment and infection prevention), users should input values for all three parameters. The app generates two stacked probability plots: one for the initial conditions and one showing the probabilities after interventions. These plots are displayed side by side for better comparisons. Moving the cursor through each plot also help to see the probability of being in a given state at each time point.

### Simulations setup and data source

For our simulations, we will use a hypothetical hospital scenario based on real world clinical data. Through a retro calculation process (available in S3 File), we determined the transition hazard rates from a published paper [15]. The paper was on retrospective cohort study of 268 adult COVID-19 patients admitted to the intensive care unit (ICU) at the University Medical Center Freiburg between February 2020 and July 2021. All of them faced risks of hospital-acquired superinfections caused by diverse pathogens during their ICU stay. For analytical simplicity, we do not distinguish between the types of superinfections. The multistate model includes the following transition states: 0-ICU stay with COVID-19 (no superinfection), 1-Acquisition of superinfection, 2-Discharge alive without superinfection, 3-Death without superinfection, 4-Discharge alive after superinfection, 5-Death after superinfection. We define the following transition-specific baseline hazard rates: λ_01_ = 0.0268, λ_02_ = 0.0143, λ_03_ = 0.0087, λ_14_ = 0.0472, λ_15_ = 0.0527. The simulations in StaViC were conducted with α = 0.5 and β = 0.5, θ = 0.3. It is important to recall that α and β = 0.5, representing a 50% improvement in treatment effectiveness, while θ = 0.3, representing a 70% reduction in the risk of acquiring a superinfection.

While in HAISim it is possible to see the mortality, the number if patient days, the change in length of stay and the reduced patient-days, for simplicity purposes, in the results we will only highlight the number of lives saved and reduced patient-days.

## Results

Fixing α and β at 0.5 resulted in 4 lives saved and −903.3 “reduced patient-days” per 268 patients, where the negative value indicates an increase in patient-days, based on Setting 1 simulations of enhanced treatment using real-world clinical data in HAISim. For “reduced patient-days,” positive values indicate the number of hospital days shortened, whereas negative values explicitly correspond to an increase in patient-days compared to baseline. The impact of the intervention decreased when α and β were adjusted to 0.7, resulting in only 2 lives saved and −433.5 reduced patient days per 268 patients. Fixing all three parameters (α, β, and θ) at 0.7 resulted in 24 lives saved and 129.3 reduced patient days per 268 patients under Setting 2, which combines enhanced therapy and infection prevention. The intervention had significantly more positive effects when all parameters were set at 0.5, saving 40 lives and 270.0 reduced patient-days per 268patients (**Table 2**).

**Table 2:** Estimated number of lives saved and patient-days reduced under different intervention scenarios in simulations using HAISim.

Settings	α	β	θ	Lives Saved^*1*^	Patient-days Reduced^*1*^
Setting 1	0.5	0.5	–	4	− 903.3
	0.7	0.7	–	2	− 433.5
Setting 2	0.5	0.5	0.5	40	270.0
	0.7	0.7	0.7	24	129.3

^*1*^Number of lives saved/ Patient-days reduced per 268 Patients

**Fig 4:**
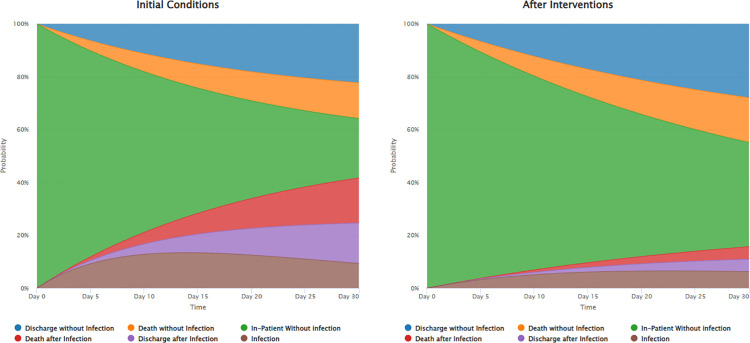
Stacked probability plots over a 30-day period using StaViC for simulations.

Using StaViC for simulations, the peak probability of superinfection decreased from 13.40% initially observed on Day 13 to 6.51% now on Day 22. A comparable decline was observed in the peak probability of death among patients who acquired a superinfection during their ICU stay, which dropped from 17.09% to 4.73%. Conversely, the peak probability of a COVID patient being discharged alive without ever experiencing superinfection increased from 22.27% to 27.91%. Although superinfection incidence and its related death probabilities declined, the probability of death without superinfection was approximately 1.25 times higher than under the initial conditions (**Fig 4**).

## Discussion

In this work, we developed two useful tools that can be exploited for different purposes ranging from clinical insights to public health decision-making. Using the two interactive and user-friendly apps combined can be of great support in implementing appropriate infection prevention and control measures in a hospital setting. HAISim simulates and evaluates the impact of various intervention strategies on hospital-acquired infections outcomes by modeling different intervention factors. StaViC helps to understand the dynamics of patients who acquire an infection after hospital admission, through the visualization of the probability of patients being in different health states of the model over time. In our study, based on a hypothetical hospital scenario using real-world clinical data, HAISim simulations incorporating only enhanced treatment of superinfection resulted in negative values for reduced patient-days when the enhanced treatment parameters (α and β) values were set to 0.5 and 0.7. The negative values of reduced patient-days suggest that, instead of dying, some COVID-19 patients who later acquired a superinfection would survive due to the enhanced treatment interventions. As shown in Table 2 under the number of lives saved, these interventions would reduce mortality. However, patients who survive because of treatment may remain longer in the superinfection state (when the undergoing the treatment) before eventually being discharged. This extended stay contributes to a longer overall length of stay, resulting in negative values for reduced patient-days. StaViC simulations also showed a number of significant implications. StaViC was used to compare pre- and post-intervention conditions under a combined strategy of 70% improved superinfection prevention and 50% enhanced treatment. This led to a substantial reduction in the probabilities of acquiring a superinfection and the associated mortality while increasing the probabilities of patients being in-patient without acquiring a superinfection, as illustrated in **Fig 4**. When taken as a whole, these changes suggest that patient trajectories have improved. However, it is important to highlight that the peak probability of death for patients who did not experience a superinfection increased by approximately 1.25 times in comparison to the initial conditions. In this hypothetical scenario, these insights could therefore assist choose and optimize interventions, while considering not only the lives saved but also aiming for optimum discharge hazard rates.

While these tools can be applied to a range of infection types, it remains essential that users clearly define the infection of interest and specify the clinical or laboratory criteria used to characterize it. Similarly, the interventions for which potential benefits are being assessed whether preventive bundles (e.g., catheter care, VAP bundles, antibiotic stewardship) or treatment-related measures should be explicitly described in any reports to ensure proper interpretation and comparability.

When trying to implement, adapt or adjust the treatment of certain nosocomial infections, several questions might arise. The direct one with a clinical relevance will be to know among all the new molecules or combinations available, which one will lead to better clinical outcomes (efficacy leading to cure and discharge or less mortality)? However, evaluating the effects on hospital length of stay, overall healthcare expenses, and mortality is equally relevant from the perspective of hospital management and resource allocation. Optimizing resource use is crucial when implementing novel therapies or infection prevention strategies because hospitalization costs due to length of stay differ between countries and healthcare systems. In general, the best new treatment to be implemented in the hospital not only should be appropriate in terms of clinical efficacy but also should align with resources optimization.

As an illustration, Bassetti et al. [16] published a review on newly approved medications by FDA (Food and Drug Administration) and EMA (European Medicines Agency) for the treatment of hospital-acquired pneumonia and ventilator-associated pneumonia. Some RCTs also revealed the effectiveness of infection prevention interventions such as enhanced cleaning and disinfection, use of antiseptic barrier caps and an environmental cleaning bundle [17–19]. If a hospital wants to implement one of the new drugs and/or implement a new intervention to reinforce infection prevention; by going from settings (with similar size) where those drugs or interventions were previously or are currently being implemented, it is possible to evaluate how it impacted the transition hazard rates of infection, death and for discharge alive after infection in these hospitals. By doing this, we can then get the values of the enhanced treatment factors (α and β) as well as that of the infection prevention factor (θ). The intervention related factors can either be obtained from published literature or through local hospital data (as shown in S4 File). Then by entering the hospital’s current transition rates in HAISim, it is possible to see the different values of α, β and θ from other hospitals with similar size, the possible mortality reduction, the number of lives saved, the change in length of stay and the number of patient-days reduced for each set of factors. Then clinicians and hospital administrators or healthcare financing experts can all have a global idea of how they could reduce the mortality but also the patient-days. Then for more clinical insights, it is possible to use the values of selected set of α, β and θ, and visualize using StaViC, the probability of patients being in different states over time after admission into the setting. It helps to assess after how many days could the patient reach the peak of the infection state or monitor the peak probability of death (in case of infection acquisition).

In their study, Hazard et al. [13] presented a multi-state modeling approach to predict the potential impact of infection prevention strategies on the burden of nosocomial infections in hospitals. They have therefore demonstrated in a concrete way that calculating the impact of an intervention (represented by θ) enables both a cost-benefit analysis of these actions as well as an evaluation of the effectiveness of the measures implemented. This approach was used for a hypothesized intervention among neonates in a study [4] in an Ethiopian hospital, where a 50% decrease in infections (θ = 0.5) lead to 101 lives saved and 1 357 reduced days-patients. Grodd et al. [20] designed an application to visualize probability plots based on constant hazards. It additionally displays the attributable mortality (AM) of an HAI and the population attributable fraction (PAF). Based on this, our version (StaViC) aims to enhance visualization while facilitating direct comparisons of interventions between groups or in simulation scenarios for different interventions.

In facilities with well-established infection prevention and control (IPC) surveillance programs, particularly those targeting specific infections, the tools developed in this study (HAISim and StaViC) may provide meaningful added value. When integrated into existing systems, they can support short-term evaluations and inform potential adaptations of ongoing surveillance strategies. This is especially relevant in settings where cohort-specific data on targeted infections are routinely collected or reported, as these tools can help translate surveillance data into actionable insights to guide and optimize IPC efforts. When using rates derived from surveillance databases, sensitivity analyses should be conducted by varying default hazard rates, and in settings where underreporting is suspected, infection rates should be increased accordingly to assess the robustness of the results. However, the validity of the findings depends on the quality and completeness of the underlying surveillance data, and misclassification or underreporting of infections may influence the estimated impact of the tools.

With the recent advances in the digitalization of healthcare, it is still crucial to maintain patient privacy and data protection [21]. The R Shiny apps developed in this work can be used without concerns about data security. First of all, it is important to point out that patients’ data are not directly entered into the apps. Rather, only pre-calculated transition rates are used. Then, no usage history is stored by the apps, so no hospital-specific transition rates are recorded. In addition, the full source codes of the apps are also included in this publication, which increases transparency and offers the option of choosing between using the online version or running the apps on a local system. The generalizability of these applications is another strength of this work. Although in this work we have developed the applications by highlighting how they can support the implementation of infection prevention and control interventions in a hospital setting, it is also possible to employ them in the context of pandemic preparedness for potential simulations. In such scenarios, rapid intervention is essential, but it is equally important to be able to assess illness transmission dynamics, anticipate healthcare system capacity constraints and optimize resource distribution. By offering a data-driven approach to evaluating intervention strategies, these applications could facilitate real-time decision-making, improve epidemic response efforts and, ultimately, inform policy decisions and resource allocation during future public health crises.

A major limitation of the study is the assumption of constant risks for transitions between states. This assumption of constant transitions served as the basis for the multistate model and, subsequently, for the R Shiny Apps that were developed. However, transition rates are often not constant in real- world situations. They may vary according to the type of infection, patient characteristics or stage of infection. These differences in transition rates can affect the generalizability of in the use of the apps, particularly in situations where dynamic transitions better reflect clinical realities. Assuming a homogeneous patient population in our study helps to focus on population-level effects of potential interventions, as the analysis relies solely on transition hazard rates as inputs. However, this assumption can also be a limitation. In cases where individual or patient-specific covariates are available, the analysis should be extended to incorporate these variables, allowing for proper adjustment for confounding through regression modeling (whether for etiologic or prognostic purposes). Such an extension would be similar to the approach by Cran *et al.* [22] exploring artificially manipulating transition intensities, with applications to sickness absence and work.

## Conclusion

In this study, two multi-state models incorporating the implementation of hypothetical interventions were developed and implemented in R Shiny applications. When used together, these technologies provide valuable tools to support the implementation of infection prevention and control measures in hospitals. Additionally, they provide a flexible framework for simulations in various scenarios, which help in strategic planning and decision-making in healthcare facilities.

## Supporting information

S1 File Supporting information File 1. (DOCX)

S2 File Supporting information File 2. (DOCX)

S3 File Supporting information File 3. (DOCX)

S4 File Supporting information File 4. (DOCX)

## Abbreviations

HAI: Hospital-acquired infection
IPC: Infection Prevention and Control
HAISim: Hospital-acquired infection Interventions Simulator
StaViC: Stacked probAbility Visualization & Comparison
RM: Reduced mortality
LoS: Length of stay
cLoS: Change in length of stay
LivSaved: Lives saved
