# Supplementary material for: Evaluating hypothetical interventions effects on hospital-acquired infection outcomes with stacked probability visualization: R Shiny apps based on a multistate modelling approach
Source: PLoS One. 2026 Mar 16;21(3):e0343837. doi: 10.1371/journal.pone.0343837 (PMC12991248; doi:10.1371/journal.pone.0343837)
Supplement: S1 File — (DOCX) [file pone.0343837.s001.docx]

**Detailed notations (for Equations 1-8)**

| **Symbol** | **Definition** |  |
| --- | --- | --- |
| *λ_ij_* | Transition hazard from state *i* to state *j* | |
| *λ^T^_ij_* | Transition hazard rate from state *i* to another sate *j* in the context of enhanced treatment | |
| θ | Disease prevention factor | |
| *α* | Effect of the enhanced treatment on discharge | |
| *β* | Effect of the enhanced treatment on Death | |
| Prob0 | Overall probability of hospital death in Default Setting | |
| Prob1 | Overall probability of hospital death in Setting 1 (Intervention 1 only) | |
| Prob2 | Overall probability of hospital death in Setting 2 (Intervention 1 + Intervention 2) | |
| LoS0 | Expected overall length of hospital stay in Default Setting | |
| LoS1 | Expected overall length of hospital stay in Setting 1 | |
| LoS2 | Expected overall length of hospital stay in Setting 2 | |
| RM1 | Reduced mortality in Setting 1 (Prob0 − Prob1) | |
| RM2 | Reduced mortality in Setting 2 (Prob0 − Prob2) | |
| cLoS1 | Change in length of stay in Setting 1 (LoS0 − LoS1) | |
| cLoS2 | Change in length of stay in Setting 2 (LoS0 − LoS2) | |
| Patient-Days1 | Total patient-days reduced in Setting 1: cLoS1 × n | |
| Patient-Days2 | Total patient-days reduced in Setting 2: cLoS2 × n | |
| LivSaved1 | Number of lives saved in Setting 1: RM1 × n | |
| LivSaved2 | Number of lives saved in Setting 2: RM2 × n | |
| n | Total number of patients in the cohort | |
