# Supplementary material for: Evaluating hypothetical interventions effects on hospital-acquired infection outcomes with stacked probability visualization: R Shiny apps based on a multistate modelling approach
Source: PLoS One. 2026 Mar 16;21(3):e0343837. doi: 10.1371/journal.pone.0343837 (PMC12991248; doi:10.1371/journal.pone.0343837)
Supplement: S2 File — (DOCX) [file pone.0343837.s002.docx]

In this supporting information file, we provide more details on the transition intensity matrix and transition probabilities of the study. This was previously demonstrated by von Cube et al. (2017) for the extended disease-death model.

In our study, we used a model with six (6) transition states.

**States:**

0-Admission into hospital

1-Hospital-acquired infections (HAI)

2-Discharged Alive after Admission (and stay in hospital setting without acquiring HAI)

3-Death after Admission (and stay in hospital setting without acquiring HAI)

4-Discharged Alive after admission followed by HAI

5-Death after Admission followed by HAI.

**Transitions hazards:**

|  | **Setting 1** | **Setting 2** |
| --- | --- | --- |
| 0 -> 1 | *λ_01_* | *θλ_01_* |
| 0 -> 2 | *λ_02_* | *λ_02_* |
| 0 -> 3 | *λ_03_* | *λ_03_* |
| 1 -> 4 | *λ^T^_14_* | *λ^T^_14_* |
| 1 -> 5 | *λ^T^_15_* | *λ^T^_15_* |

For Setting 1: *λ_0_*= *λ_01_* + *λ_02_* + *λ_03_* and *λ_1_*= *λ^T^_14_* + *λ^T^_15_*;

For Setting 2: *λ_0_*= *θλ_01_* + *λ_02_* + *λ_03_* and *λ_1_*= *λ^T^_14_* + *λ^T^_15_*.

**Transition Intensity Matrix (Q_1_) and Transition Probability Matrix (P_1_) for Setting 1**

**
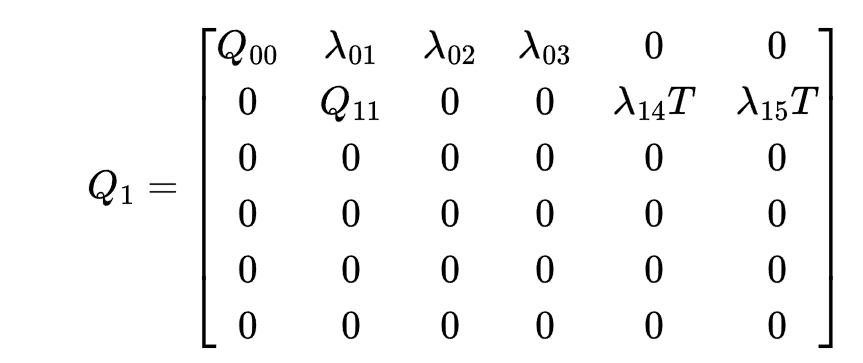
**

**Note:** *λ_15_T***=***λ^T^_15_* and *λ_14_T***=***λ^T^_14_*

The Transition Probability Matrix represents the probabilities of transitioning from one state to another in a given time interval 𝑡 t. It is derived from the matrix exponential: $P(t)=e^{Qt}$


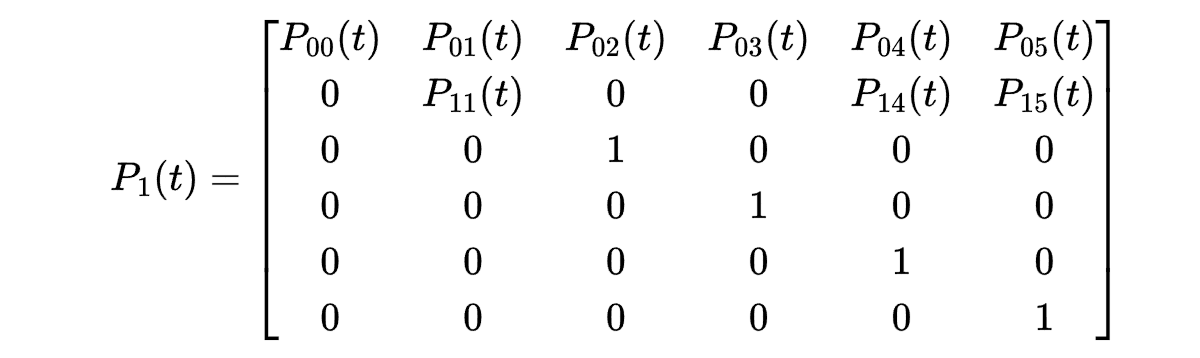

The specific transition probabilities can be expressed as follows:

$$P_{00}\left( 0, t \right)=e^{-\lambda_{0}\cdot t}$$

$$P_{01}\left( 0, t \right)=\frac{\lambda_{01}}{\lambda_{1}- \lambda_{0}}\left( e^{-\lambda_{0}\cdot t}-e^{-\lambda_{1}\cdot t} \right)$$

$$P_{02}\left( 0, t \right)=\frac{\lambda_{02}}{\lambda_{0}}\left( 1-e^{-\lambda_{0}\cdot t} \right)$$

$$P_{03}\left( 0, t \right)=\frac{\lambda_{03}}{\lambda_{0}}\left( 1-e^{-\lambda_{0}\cdot t} \right)$$

$$P_{04}\left( 0, t \right)=\frac{\lambda_{14}^{T}}{\lambda_{1}}\left( 1-P_{00}\left( 0,t \right)-P_{01}\left( 0,t \right)-P_{02}\left( 0,t \right)-P_{03}\left( 0.t \right) \right)=\frac{\lambda_{01}\cdot\lambda_{14}^{T}}{\lambda_{0}\cdot\lambda_{1}}-\frac{\lambda_{01}\cdot\lambda_{14}^{T}}{\lambda_{0}\cdot\left( \lambda_{1}- \lambda_{0} \right)}\left( e^{-\lambda_{1}\cdot t}-e^{-\lambda_{0}\cdot t} \right)$$

$$P_{05}\left( 0, t \right)=\frac{\lambda_{15}^{T}}{\lambda_{1}}\left( 1-P_{00}\left( 0,t \right)-P_{01}\left( 0,t \right)-P_{02}\left( 0,t \right)-P_{03}\left( 0.t \right) \right)=\frac{\lambda_{01}\cdot\lambda_{15}^{T}}{\lambda_{0}\cdot\lambda_{1}}-\frac{\lambda_{01}\cdot\lambda_{15}^{T}}{\lambda_{0}\cdot\left( \lambda_{1}- \lambda_{0} \right)}\left( e^{-\lambda_{1}\cdot t}-e^{-\lambda_{0}\cdot t} \right)$$

For every time t, we have: $P_{00}\left( 0, t \right)+ P_{01}\left( 0, t \right)+ P_{02}\left( 0, t \right)+ P_{03}\left( 0, t \right)+ P_{04}\left( 0, t \right)+ P_{05}\left( 0, t \right)=1$

**Transition Intensity Matrix (Q_2_) and Transition Probability Matrix (P_2_) for Setting 2**

The transition intensity matrix is expressed as follows:

**
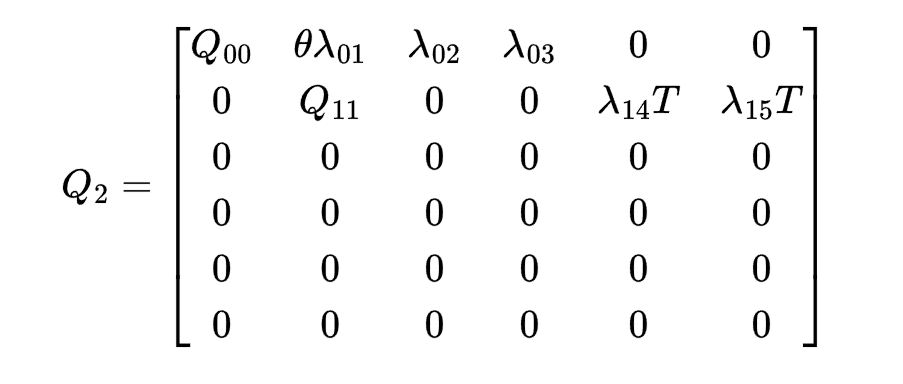
**

**Note :** *λ_15_T***=***λ^T^_15_* and *λ_14_T***=***λ^T^_14_*

The Transition Probability Matrix is also expressed as:


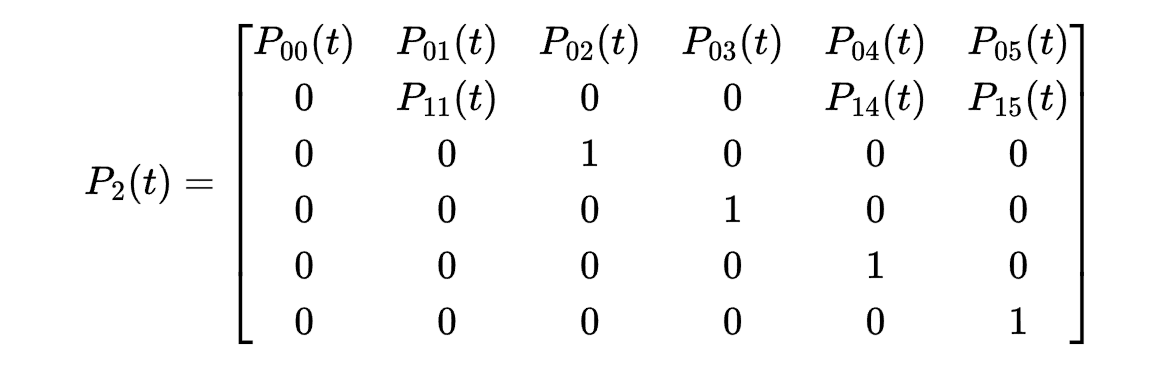

The specific transition probabilities for Setting 2 can be expressed as follows:

$$P_{00}\left( 0, t \right)=e^{-\lambda_{0}\cdot t}$$

$$P_{01}\left( 0, t \right)=\frac{{\theta\lambda}_{01}}{\lambda_{1}- \lambda_{0}}\left( e^{-\lambda_{0}\cdot t}-e^{-\lambda_{1}\cdot t} \right)$$

$$P_{02}\left( 0, t \right)=\frac{\lambda_{02}}{\lambda_{0}}\left( 1-e^{-\lambda_{0}\cdot t} \right)$$

$$P_{03}\left( 0, t \right)=\frac{\lambda_{03}}{\lambda_{0}}\left( 1-e^{-\lambda_{0}\cdot t} \right)$$

$$P_{04}\left( 0, t \right)=\frac{\lambda_{14}^{T}}{\lambda_{1}}\left( 1-P_{00}\left( 0,t \right)-P_{01}\left( 0,t \right)-P_{02}\left( 0,t \right)-P_{03}\left( 0.t \right) \right)=\frac{{\theta\lambda}_{01}\cdot\lambda_{14}^{T}}{\lambda_{0}\cdot\lambda_{1}}-\frac{{\theta\lambda}_{01}\cdot\lambda_{14}^{T}}{\lambda_{0}\cdot\left( \lambda_{1}- \lambda_{0} \right)}\left( e^{-\lambda_{1}\cdot t}-e^{-\lambda_{0}\cdot t} \right)$$

$$P_{05}\left( 0, t \right)=\frac{\lambda_{15}^{T}}{\lambda_{1}}\left( 1-P_{00}\left( 0,t \right)-P_{01}\left( 0,t \right)-P_{02}\left( 0,t \right)-P_{03}\left( 0.t \right) \right)=\frac{{\theta\lambda}_{01}\cdot\lambda_{15}^{T}}{\lambda_{0}\cdot\lambda_{1}}-\frac{\theta\lambda_{01}\cdot\lambda_{15}^{T}}{\lambda_{0}\cdot\left( \lambda_{1}- \lambda_{0} \right)}\left( e^{-\lambda_{1}\cdot t}-e^{-\lambda_{0}\cdot t} \right)$$

$P_{00}\left( 0, t \right)+ P_{01}\left( 0, t \right)+ P_{02}\left( 0, t \right)+ P_{03}\left( 0, t \right)+ P_{04}\left( 0, t \right)+ P_{05}\left( 0, t \right)=1$
