# Supplementary material for: Evaluating hypothetical interventions effects on hospital-acquired infection outcomes with stacked probability visualization: R Shiny apps based on a multistate modelling approach
Source: PLoS One. 2026 Mar 16;21(3):e0343837. doi: 10.1371/journal.pone.0343837 (PMC12991248; doi:10.1371/journal.pone.0343837)
Supplement: S3 File — (DOCX) [file pone.0343837.s003.docx]

In this supporting information file, we show how transition hazard rates can be calculated by directly using data reported in published articles where information on length of stay and mortality risks are available.

**The Extended Illness-Death Model with six states**

**
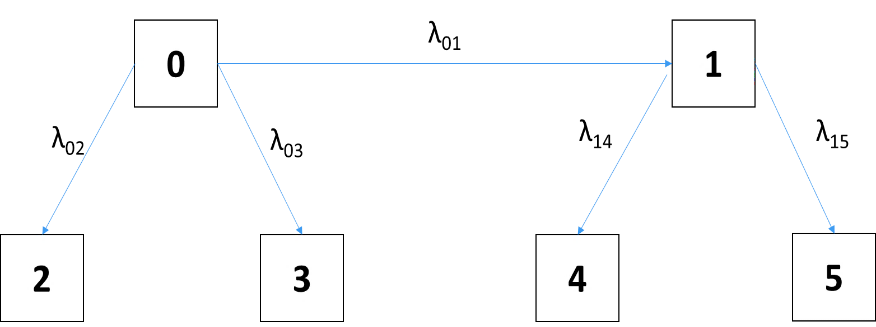
**

**States:** 0 = Admission, 1 = HAI, 2 = Discharge from Admission,

3 = Death from Admission, 4 = Discharge after HAI, 5 = Death after HAI.

**Transitions occur between states with constant hazard rates**

λ₀₁: acquisition of HAI, λ₀₂: discharge without HAI, λ₀₃: death without HAI, λ₁₄: discharge after HAI,

λ₁₅: death after HAI. λ_0_=λ_01_+λ_02_+λ_03_ and λ_1_​=λ_14_​+λ_15​_

**Formulas**

- Probability of acquiring HAI (0 to 1): $P=\frac{\lambda01}{\lambda01+\lambda02+\lambda03}= \frac{\lambda01}{\lambda0}$
- Mortality risk from Admission (given that they didn’t get HAI) : Mo $=\frac{\lambda03}{\lambda02+\lambda03}$
- Mortality risk after HAI: M_1_ $=\frac{\lambda15}{\lambda14+\lambda15}= \frac{\lambda15}{\lambda1}$
- Length of stay in Admission (Sojourn time in Admission state): LoS_0_​$=\frac{1}{\lambda01+\lambda02+\lambda03}= \frac{1}{\lambda0}$
- Length of stay in HAI (Sojourn time in HAI state): LoS_1_​$=\frac{1}{\lambda14+\lambda15}= \frac{1}{\lambda1}$

**Important note:** Several publications provide only the combined “Admission + infection” sojourn time. Using the combined sojourn time without decomposition will bias transition hazard estimates. When LoS₁ is not directly available, it can be computed by subtracting the admission-state sojourn time from the reported total LoS for infected patients: LoS_1_​=LoS_total_ _(infected)_​−LoS_0​_.

**Retro-calculation of Transition hazard rates:**

(Recover component hazards from 𝑃, 𝑀_0_, 𝑀_1_, LoS_0_, LoS_1_, M_0_, M_1_, LoS_0_, and LoS1)

**λ_0_ ​**$\boldsymbol{=}\frac{\mathbf{1}}{\mathbf{LoS}\mathbf{₀}\mathbf{}}$

**λ_01_= P · λ_0_ =** $\frac{\mathbf{P}}{\mathbf{LoS}\mathbf{₀}\mathbf{}}$

λ₀₂ + λ₀₃ = λ₀ − λ₀₁ = λ₀(1 − P);

λ_03_​= M₀ · λ₀ (1 − P) therefore **λ_03_ =** $\frac{\boldsymbol{M₀ \cdot(1 - P)}}{\mathbf{LoS}\mathbf{₀}\mathbf{}\mathbf{}}$

​λ_02_ = λ_0_ - λ_01_​ ​- λ_03_ = λ₀(1 − P) - λ_03_ = λ₀(1 − P) ​- M₀ · λ₀ (1 − P) ;

λ₀₂ = (1 − M₀) · λ₀ (1 − P) therefore **λ_02_ =** $\frac{\boldsymbol{(1 - M₀) \cdot(1 - P)}}{\mathbf{LoS}\mathbf{₀}\mathbf{}\mathbf{}}$

**λ_1_ ​**$\boldsymbol{=}\frac{\mathbf{1}}{\mathbf{LoS}\mathbf{₀}\mathbf{}}$

λ_15_ = M_1​_λ_1_ therefore **λ_15_ ​**$\boldsymbol{=}\frac{M\mathbf{₁}}{\mathbf{LoS}\mathbf{₁}\mathbf{}}$

λ_14_​= λ_1_ - λ_15_ = $\frac{1}{\mathrm{LoS}\mathbf{₁}}$ - $\frac{M\mathbf{₁}}{\mathrm{LoS}\mathbf{₁}}$ and therefore **λ_14_ ​**$\boldsymbol{=}\frac{\mathbf{1-M}\mathbf{₁}}{\mathbf{LoS}\mathbf{₁}\mathbf{}}$

**Application to data extracted from Table 1 in the publication from *Lösslein et al., 2025***

(<https://doi.org/10.1186/s12879-025-10983-7>)

**Extracted data (From Table 1 of the paper):**

Total N = 268

Total Number with HAI: 144

Total Number with No-HAI: 124

Deaths among No-HAI group: 47

Deaths among HAI group: 76

LoS_0_ = 20 days

LoS_1_ = 30 - 20 _=_ 10 days*

***In the paper, LoS_1_ was not directly reported therefore we computed it by doing LoS_1_​=LoS_total_ _(infected)_​-LoS_0_ _​_as explained earlier.

**Determining Mortality risks and Probability of infection:**

P = 144/268 = 0.5373

M_0_ = 47/124 = 0.3790

M_1_ = 76/144 = 0.5278

**Determining the Transition hazard rates:**

λ_0_ = 1/LoS_0_ = 1/20 = 0.05 day^−1^.​

λ_1_​= 1/LoS_1​_ = 1/10 = 0.1 day^−1^

λ_01_​ ​​= P / LoS₀ = 0.5373/20 = 0.0268 day^−1^

λ₀₂ = (1 − M₀) · (1 − P) / LoS₀ = (1 − 0.3790) · (1 − 0.5373) / 20 = 0.0143 day^−1^

λ₀₃ = M₀ · (1 − P) / LoS₀ ​​= 0.3790 · (1 − 0.5373) / 20 ​​= 0.0087 day^−1^

λ₁₄ = (1 − M₁) / LoS₁ = (1 − 0.5278) / 10 = 0.0472 day^−1^

λ₁₅ = M₁ / LoS₁ ​​= 0.5278 /10 ​​=0.0527 day^−1^
