# Supplementary material for: Evaluating hypothetical interventions effects on hospital-acquired infection outcomes with stacked probability visualization: R Shiny apps based on a multistate modelling approach
Source: PLoS One. 2026 Mar 16;21(3):e0343837. doi: 10.1371/journal.pone.0343837 (PMC12991248; doi:10.1371/journal.pone.0343837)
Supplement: S4 File — (DOCX) [file pone.0343837.s004.docx]

**Calculating Intervention-Related Factors (α, β, θ) from Local Hospital Data**

**Overview**

This supplementary file illustrates how hospitals can calculate intervention-related factors using locally collected pre- and post-intervention data. These factors quantify the effectiveness of infection prevention and treatment interventions in reducing hospital-acquired infection (HAI) incidence, HAI-related mortality, and improving discharge rates, and can be incorporated into the multistate model described in the main manuscript.

It is important to mention that, this is also based on all the assumptions in our model as described in the methodology and using Equations 1-8 of the main manuscript.

**1. Data Requirements**

For both the **baseline period** (previous or no intervention) and the **post-intervention period**, collect the following:

**All hospitalized patients:**

- Total number of admissions (n)
- Total patient-days
- Number of discharges alive
- Number of deaths

**Patients who developed HAI:**

- Number of HAI cases
- Number of discharges alive among HAI patients
- Number of deaths among HAI patients
- Total patient-days for HAI patients

**
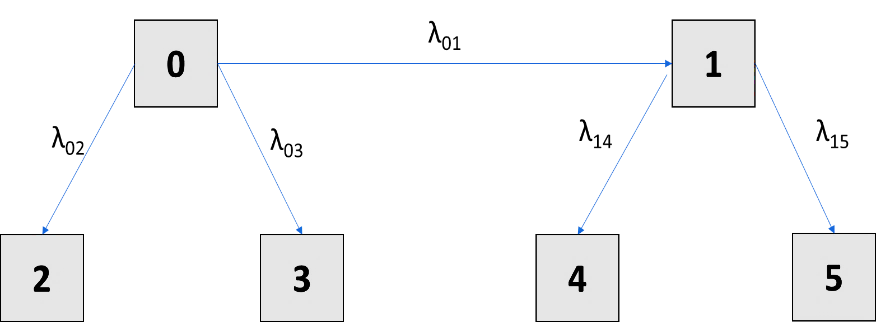
**

**States:** 0 = Admission, 1 = HAI, 2 = Discharge from Admission, 3 = Death from Admission, 4 = Discharge after HAI, 5 = Death after HAI.

**Transitions occur between states with constant hazard rates:** λ₀₁: acquisition of HAI, λ₀₂: discharge without HAI, λ₀₃: death without HAI, λ₁₄: discharge after HAI, λ₁₅: death after HAI. λ_0_=λ_01_+λ_02_+λ_03_ and λ_1_​=λ_14_​+λ_15​_

**2. Estimating Transition-Specific Hazard Rates**

**2.1 Baseline Period (Default Setting)**

$$\lambda01=\frac{Number of HAI cases}{Total patient-days in State 0}$$

$$\lambda02=\frac{Number of discharges without HAI}{Total patient-days in State 0}$$

$$\lambda03=\frac{Number of deaths without HAI}{Total patient-days in State 0}$$

$$\lambda14=\frac{Number of discharges with HAI}{Total patient-days in State 1}$$

$$\lambda15=\frac{Number of deaths with HAI}{\mathrm{Total}\mathrm{patient}-days in State 1}$$

**2.2 Post-Intervention Period**

Similarly calculate the same hazard rates using post-intervention data, denoted as λ′_01_, λ′_02_, λ′_03_, λ′_14_ and λ′_15_.

Before proceeding it is important to recall the following:
Treatment interventions lead to: λ^T^_14_ = α(λ_14_ – λ_02_) + λ_02_ and λ^T^_15_ = β(λ_15_ - λ_03_) + λ_03_

Prevention interventions lead to: λ’_01_ = θλ_01_

With λ′_14_ = λ^T^_14_ and λ′_15_ = λ^T^_15._

**3. Calculating Intervention-Related Factors**

**3.1 Prevention Factor (θ)**

θ represents the effect of infection prevention interventions in reducing HAI incidence:

θ = λ′_01_ / λ_01_

**Interpretation:** θ = 1: no prevention effect; θ = 0.5: 50% reduction in HAI incidence; θ = 0: complete prevention of HAI

**Example:**
If λ_01_ = 0.020 and λ’_01_ = 0.012, θ=0.012/ 0.020=0.6 ​=0.6, Indicating a 40% reduction in HAI incidence.

**3.2 Treatment Effect on Discharge (α)**

α is the effect of enhanced treatment on discharge rates among infected patients:

*λ^T^_14_* = *α*(*λ_14_* – *λ_02_*) + *λ_02_* ⇒ α=λ′_14_ − λ_02_ / λ_14_​−λ_02_​

**Interpretation:**

α = 1: No improvement in discharge rates (no treatment effect)

α = 0.5: 50% improvement toward non-infected discharge rates

α = 0: Complete normalization of discharge rates to non-infected levels

**Example:** If λ₁₄ = 0.080, λ₀₂ = 0.120, and λ'₁₄ = 0.100 per patient-day:

α= (0.100−0.120) / (0.080−0.120) = 0.5

This indicates a Treatment leading to 50% improvement in discharge rates among infected patients.

**3.3 Treatment Effect on Mortality (β)**

β is the effect of enhanced treatment on death rates among infected patients:

*λ^T^_15_* = β (*λ_15_* – *λ_03_*) + *λ_03_* ⇒ β =λ′_15_ - λ_03_ / λ_15_​-λ_03_

**Interpretation:**

β = 1: No reduction in mortality (no treatment effect)

β = 0.5: 50% reduction in excess mortality due to HAI

β = 0: Complete elimination of excess mortality (mortality equals non-infected level)

**Example:** If λ_15_ = 0.030, λ03 = 0.10, and λ’_15_= 0.020 per patient-day:

α= (0.020−0.010) / (0.030−0.010) = 0.5

This indicates a treatment leading to a 50% reduction in excess HAI-related mortality.
